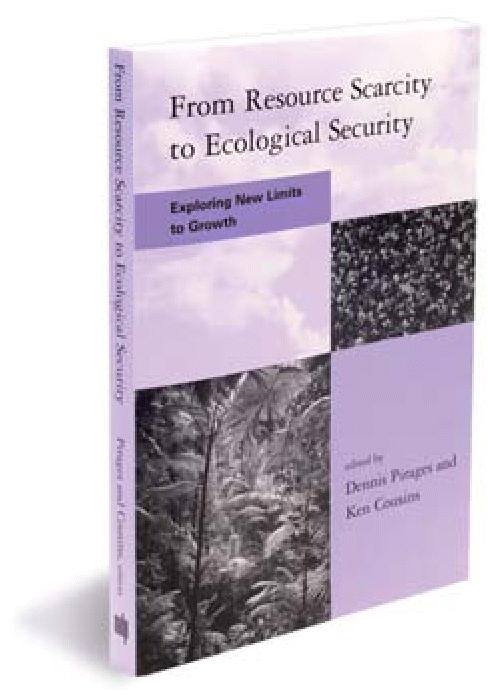# From Resource Scarcity to Ecological Security: Exploring New Limits to Growth

**Published:** 2006-03

**Authors:** Angus Cook

**Affiliations:** Angus Cook is the director of the Ecology and Health group at the School of Population Health, and an associate research fellow at the Asthma and Allergy Research Institute, The University of Western Australia, Perth, Australia. His primary research interests relate to the interrelationships between human disease and ecosystem disruption.

Edited by Dennis Pirages and Ken Cousins

Cambridge, MA:MIT Press, 2005. 268 pp. ISBN: 0-262-16231-8, $60

In the face of declining resource availability coupled with increasing consumer demand, the publication of *From Resource Scarcity to Ecological Security: Exploring New Limits to Growth* is a welcome addition to the literature. Over 30 years have passed since the MIT Press published the groundbreaking *The Limits of Growth*, and this new book reminds us of the urgent need to manage our unprecedented growth—and our often unreasonable demands.

This collection of essays has successfully captured the most pressing issues arising from regional and global scarcity. The challenges faced by our regional and global communities—including overpopulation, climatic disruption, and loss of ecosystem services—indeed present a complex theme, but all have been handled with compelling analyses by the authors.

The theoretical basis for the book is set in the first two chapters, which elaborate on predictions for growth in the world community, the likely resource requirements of current and future global inhabitants, and the extent to which ecosystems can continue to reasonably fulfill these needs. The refinements of demographic predictions, including the consequences of changing age structures in developed and developing countries, offer an important update on the kinds of futures that human populations might expect.

The imbalance between demand and supply is becoming particularly apparent in relation to food and water security, and these topics are thoughtfully explored by Conca (“Global Water Prospects”) and Cohen (“Food Policy: Underfed or Overfed?”). The ecological and sociopolitical drivers influencing the provision of clean water and adequate food supplies are cogently described by both authors: In each case, their analyses argue against overly simplistic interpretations of the resource problems confronting many societies.

The impact of human resource use decisions cannot be dissociated from the toll taken on ecosystems, and these consequences are explored with the context of biodiversity. Marchak’s description (“Forest Degradation, the Timber Trade, and Tropical-Region Plantations”) provides a particularly sobering case of how market-driven initiatives—especially those with a global reach—have resulted in widespread degradation and the loss of tropical timber species.

Although resource use is a topic that has been explored previously, many of the arguments in this book are strengthened by provision of a range of plausible solutions, with acknowledgement that multiple approaches are needed, including those driven by technological, policy, and community-based innovations. For example, strategies to provide renewable energy are explored in detail and provide possible resolutions to the fuel-driven dilemmas that most modern communities are forced to confront.

The essays that deviate slightly from the direction of the remainder of the book are those on global climate change. There is no question that this problem impinges on many of the scarcity and sustainability issues examined elsewhere, and the implications of climatic variability are well synthesized by the authors. However, the emphasis of these chapters, and the form of the management solutions suggested, might have benefited from better harmonization with the other elements of the book.

In the concluding chapter, “Twenty-nine Days: Responding to a Finite World,” Cousins provides an appraisal of the ecological imbalances identified in the preceding chapters as well as an integrated framework within which some of these problems may be conceptualized and addressed. As our societies increasingly grapple with regional and global limits of growth, this book will help us define a future trajectory from scarcity to security.

## Figures and Tables

**Figure:**